# A Novel RT-LAMP for the Detection of Different Genotypes of Crimean–Congo Haemorrhagic Fever Virus in Patients from Spain

**DOI:** 10.3390/ijms24076411

**Published:** 2023-03-29

**Authors:** Begoña Febrer-Sendra, Pedro Fernández-Soto, Juan García-Bernalt Diego, Beatriz Crego-Vicente, Anabel Negredo, Juan Luis Muñor-Bellido, Moncef Belhassen-García, María Paz Sánchez-Seco, Antonio Muro

**Affiliations:** 1 Infectious and Tropical Diseases Research Group (e-INTRO), Biomedical Research Institute of Salamanca-Research Centre for Tropical Diseases at the University of Salamanca (IBSAL-CIETUS), Faculty of Pharmacy, University of Salamanca, 37007 Salamanca, Spain; 2 Laboratorio de Arbovirus, Centro Nacional de Microbiología, CIBER de Enfermedades Infecciosas (Instituto de Salud Carlos III), 28222 Madrid, Spain; 3 Microbiology and Parasitology Service, Complejo Asistencial Universitario de Salamanca, University of Salamanca, 37007 Salamanca, Spain; 4 Internal Medicine Service, Infectious Diseases Section, Complejo Asistencial Universitario de Salamanca, University of Salamanca, 37007 Salamanca, Spain

**Keywords:** Crimean–Congo haemorrhagic fever virus, CCHFV, RT-LAMP, genotypes, Spain

## Abstract

Crimean–Congo haemorrhagic fever (CCHF) is a potentially lethal tick-borne viral disease with a wide distribution. In Spain, 12 human cases of CCHF have been confirmed, with four deaths. The diagnosis of CCHF is hampered by the nonspecific symptoms, the high genetic diversity of CCHFV, and the biosafety requirements to manage the virus. RT-qPCR and serological tests are used for diagnosis with limitations. Reverse-transcription loop-mediated isothermal amplification (RT-LAMP) could be an effective alternative in the diagnosis of the disease. However, none of the few RT-LAMP assays developed to date has detected different CCHFV genotypes. Here, we designed a RT-LAMP using a degenerate primer set to compensate for the variability of the CCHFV target sequence. RT-LAMP was performed in colorimetric and real-time tests on RT-qPCR-confirmed CCHF patient samples notified in Spain in 2020 and 2021. Urine from an inpatient was analysed by RT-LAMP for the first time and compared with RT-qPCR. The amplicons obtained by RT-qPCR were sequenced and African III and European V genotypes were identified. RT-LAMP amplified both genotypes and was more sensitive than RT-qPCR in urine samples. We have developed a novel, rapid, specific, and sensitive RT-LAMP test that allows the detection of different CCHFV genotypes in clinical samples. This pan-CCHFV RT-LAMP detected viral RNA for the first time in urine samples. It can be easily performed as a single-tube isothermal colorimetric method on a portable platform in real time and without the need for expensive equipment, thus bringing molecular diagnostics closer to rural or resource-poor areas, where CCHF usually occurs.

## 1. Introduction

Crimean–Congo haemorrhagic fever (CCHF) is a tick-borne viral haemorrhagic disease caused by Crimean–Congo haemorrhagic fever virus (CCHFV) belonging to the genus Orthonairovirus, family Nairoviridae [1]. The virus is widely distributed and maintained in a natural zoonotic cycle among ticks and a wide range of birds, and wild and domestic animals. Humans become infected by bites of CCHFV-infected Ixodid ticks, mainly species of the genus Hyalomma, and/or by direct contact with blood, body fluids, or tissues of both viraemic patients or animals [2,3]. Nosocomial outbreaks, including via aerosol generation, have also been reported [4,5]. In humans, the clinical course of the CCHF varies from asymptomatic to severe haemorrhagic and fatal cases, with a very high mortality rate ranging between 5% and 30%, and up to 40% or even more, depending on reports of cases from different countries [1,6,7].

CCHFV is an enveloped, negative-sense, single-stranded RNA virus with a trisegmented genome consisting of small (S), medium (M) and large (L) segments encapsidated by the nucleoprotein (NP), plus the RNA-dependent RNA polymerase (RdRp), to initiate transcription and genome replication in the host cell. In these three segments, sequence divergence among different viral strains was found to be 20%, 31% and 22%, respectively: this represents the highest degree of sequence diversity of any arthropod-borne virus [2,8]. According to phylogenetic analysis of complete and partial S segment sequences, seven lineages of CCHFV have been distinguished and correlated with the geographical site of virus isolation: Africa 1 (genotype I), Africa 2 (genotype II) and Africa 3 (genotype III); Asia 1 (genotype IVa) and Asia 2 (genotype IVb); Europe 1 (genotype V) and Europe 2 (genotype VI) [9].

CCHFV is the most geographically widespread tick-borne virus affecting humans, having been reported in Africa, Asia (particularly the Middle East), and southeast Europe. In recent years, this area has expanded to southwest Europe, including France, Portugal and Spain [2,10]. In Spain, CCHFV was first detected in 2010 in *Hyalomma lusitanicum* ticks collected from red deer in Cáceres province: a small amplified region of the genome within the S segment showed 98% similarity with sequences recorded for CCHFV in Mauritania and Senegal, on the western coast of Africa [11]. Later studies in the same geographical area detected the CCHFV again in *Hyalomma* spp. ticks collected from ruminants [12,13] and the phylogenetic studies of the isolates revealed a close relationship with African genotype III identified in West Africa. Recently, a survey carried out in 20 locations throughout southwestern Spain detected CCHFV in both *H. lusitanicum* and *Dermacentor marginatus* ticks collected from red deer and wild boar. Viral RNA isolates grouped with genotypes IV (Africa III clade) and V (Europe I clade) [14]. More recently, a comprehensive survey in five regions in central and southwestern Spain in ticks—mostly in *Hyalomma lusitanicum*—detected the circulation of several CCHFV genotypes, including viruses belonging to genotype III (Africa III clade), genotype I (Africa I clade) and the newly proposed Africa IV clade [15].

The aforementioned studies carried out in Spain draw attention to the importance of *Hyalomma* ticks and wild ruminants in the circulation of CCHFV, the presence of different viral genotypes, the possibility of new reassortments, as well as the potential risk of transmission to the human population. Considering that CCHFV causes a potentially lethal disease in humans, and the recent marked increase in the incidence and geographical range of CCHF in southern European countries [1], particularly in new areas such as Spain [10], CCHF is considered a major infectious disease threat spreading in Europe. Thus, CCHFV was listed as a priority pathogen for research and development by the WHO [16].

In Spain, from 2013 to 2022, 12 human cases of CCHF were confirmed, with four deaths. The first case was retrospectively confirmed by PCR in a woman who developed symptoms in 2013 [17]. In 2016, the first two autochthonous CCHF cases were reported: a patient referring with tick bite (who died on the ninth day of illness) and a secondary case in a health-care worker who had close contact with the former during hospital admission [10]. By 2021, 10 more cases with three deaths were notified [15] and two additional cases (one lethal) were recently documented in 2022 [18]. In human cases where complete sequence information on CCHFV is available, it has been detected that African genotype III is distributed in Africa [8], a virus with genetic reassortment because of L and M segments group within African genotype III, and the S segment has shown homology with African genotype IV [19] and European genotype V [20]. Thus, data on viral strains identified from human cases and tick species in Spain show high genetic variability, suggesting repeated introductions from different origins, including Africa and Eastern Europe [20].

The diagnosis of CCHF is hampered by the nonspecific symptoms, the high genetic diversity of CCHFV, and the biosafety requirements to manage a virus of high biological risk. A number of laboratory assays exist, and the choice of one of them depends on the stage of the disease, the specimens available, and the validity of the test for the CCHFV strains circulating in the suspected region of exposure [21,22]. Serological methods are most likely to be useful after the first week of illness and they are less impacted by minor genomic variations. However, false-negative results in serological tests are likely during the early acute phase of the disease, so molecular methods are preferred [23].

Actually, a number of nested reverse-transcription polymerase chain reaction (nested RT-PCR) and real-time quantitative reverse-transcription polymerase chain reaction (RT-qPCR) for CCHFV RNA detection have been described [10,24,25,26,27,28,29,30,31,32]. However, RT-qPCR platforms and associated PCR commercial kits are still expensive and technically complex, thus limiting their use for point-of-care diagnosis. To overcome such drawbacks, isothermal nucleic acid amplification assays have been widely developed and improved for use, such as point-of-care testing [33,34], specially loop-mediated isothermal amplification (LAMP) assay [35]. In combination with a reverse transcriptase, LAMP can also be used for RNA amplification (RT-LAMP) [36]. RT-LAMP has several advantages making it attractive for diagnosis of infectious diseases, such as rapidity, high specificity and sensitivity. The effectiveness of this method has already been demonstrated for the detection of a variety of pathogenic RNA viruses, for example, swine flu H1N1 [37], chikungunya virus [38,39], West Nile virus [40,41], and Japanese encephalitis virus [42], as well as those causing haemorrhagic fevers such as dengue virus [43,44,45], Ebola virus [46,47], Rift Valley virus [48,49,50], and yellow fever virus [51]. Regarding CCHFV detection, some RT-LAMP assays have also been developed [52,53,54]. However, none of these RT-LAMP assays has been able to detect different CCHFV genotypes.

In the present study, we developed a novel RT-LAMP assay for colorimetric and real-time potential detection of different CCHFV genotypes. We evaluated RT-LAMP in clinical samples from patients with CCHF notified in Spain in 2020 and 2021. Additionally, for the first time, RT-LAMP was tested and compared with RT-qPCR in the detection of CCHFV RNA in plasma and urine samples.

## 2. Results

### 2.1. RT-PCR F3-B3 Verification of Target and Sensitivity

**Figure 1:**
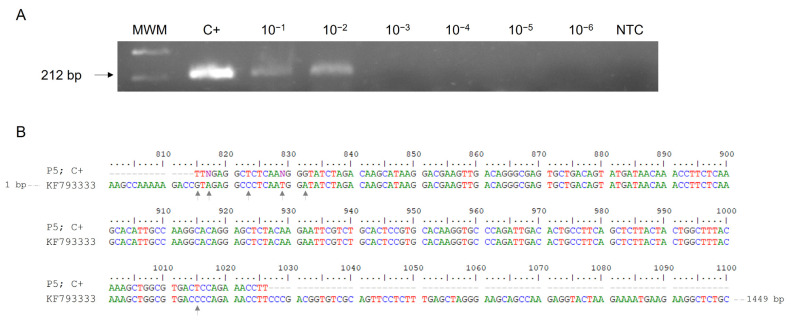
RT-PCR verification, sensitivity and sequencing using outer primers F3 and B3 for CCHFV RNA amplification. (**A**) Verification and detection limit of RT-PCR F3-B3. Lane MWM, molecular weight marker (100 bp Plus Blue DNA Ladder); lane C+, RNA isolate from patient P5 used as positive control; lanes 10^−1^–10^−6^, 10-fold serial dilutions of C+; lane NTC, non-template control (RNase-free water instead of RNA). (**B**) Comparison of 212 bp amplicon obtained (P5; C+) with partial sequence of Daral 2012 strain (GenBank KF793333). Grey arrows indicate different nucleotides in the sequence.

The expected RT-PCR 212 bp product from CCHFV RNA was successfully obtained using outer primers F3 and B3. Regarding the sensitivity, the minimum amount of CCHFV RNA detectable by one-step RT-PCR F3-B3 was 50 pg/µL (Figure 1A). Subsequent sequencing results of the 212 bp amplicon showed partial 98.08% identity with Daral 2012 strain (GenBank KF793333) of African origin (Mali) belonging to African genotype III (Figure 1B).

### 2.2. Establishing the RT-LAMP Assay: Sensitivity and Specificity

**Figure 2:**
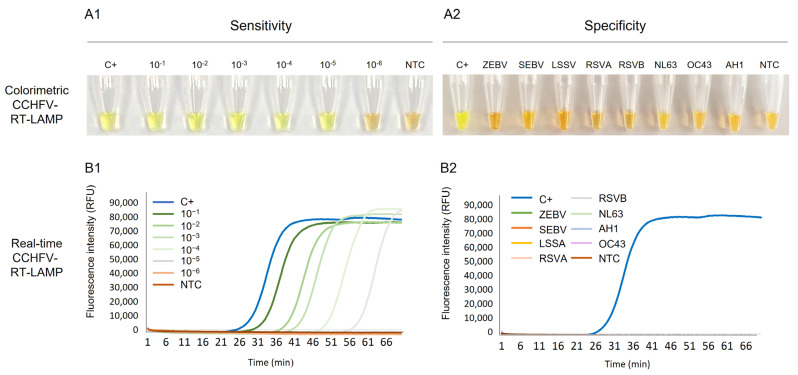
Establishing the RT-LAMP assay: sensitivity and specificity assessments for CCHFV RNA detection. (**A1**) Colorimetric RT-LAMP verification and sensitivity assessment. Lane C+, RNA-positive control (5 ng/µL); Lanes 10^−1^ to 10^−6^: 10-fold dilutions of CCHFV RNA sample used as positive control (C+); lane NTC, non-template control (ultra-pure water as template). (**A2**) Specificity assessment of colorimetric RT-LAMP. Lane C+, RNA-positive control; lanes ZEBV, SEBV, LSSV, RSVA, RSVB, NL63, OC43 and AH1: RNA from haemorrhagic viruses (Zaire Ebola virus, Sudan Ebola virus, Lassa virus) and other respiratory viruses (respiratory syncytial virus A, respiratory syncytial virus B, coronavirus NL63, coronavirus OC43 and influenza A H1, respectively. Lane NTC, non-template control (ultra-pure water as template). (**B1**). Real-time RT-LAMP verification and sensitivity assessment. Lane C+, RNA-positive control (5 ng/µL); Lanes 10^−1^ to 10^−6^: 10-fold dilutions of CCHFV RNA sample used as positive control (C+); lane NTC, non-template control (ultra-pure water as template). (**B2**) Specificity assessment of real-time RT-LAMP. Lane C+, RNA-positive control; lanes ZEBV, SEBV, LSSV, RSVA, RSVB, NL63, OC43 and AH1: RNA from haemorrhagic viruses (Zaire Ebola virus, Sudan Ebola virus, Lassa virus) and other respiratory viruses (respiratory syncytial virus A, respiratory syncytial virus B, coronavirus NL63, coronavirus OC43 and influenza A H1, respectively). Lane NTC, non-template control (ultra-pure water as template). RFU, relative fluorescence units.

Colorimetric RT-LAMP. Visual detection of RT-LAMP assays is shown in Figure 2A. The complete set of six primers designed for RT-LAMP worked well using the reaction conditions tested, and the successful amplification of CCHFV RNA could be clearly visualised by the naked eye by inspecting the colour change: positive results turned green, whereas non-template control remained orange as negative. The sensitivity of colorimetric RT-LAMP was 50 fg/µL, 10^3^ times more sensitive than the RT-PCR using outer primers F3-B3 (Figure 2(A1)). Regarding the specificity, only CCHFV RNA was amplified (green tubes) and RNA samples from other viruses tested were never amplified (orange tubes), demonstrating no cross-amplification (Figure 2(A2)).

Real-time RT-LAMP. Detection of CCHFV RNA by real-time RT-LAMP is shown in Figure 2B. We also obtained amplification of CCHFV RNA, as for colorimetric RT-LAMP, with a time to positivity (Tp) at 24 min. When testing 10-fold serial dilutions of the RNA sample used as C+, the analytical sensitivity was 50 fg/µL, identical to that obtained in colorimetric RT-LAMP assay. As can be observed, the higher the RNA dilution, the longer the amplification time (Figure 2(B1)). Furthermore, no RNA isolates from other viruses were amplified, thus indicating the high specificity of the established RT-LAMP assay (Figure 2(B2)).

### 2.3. Clinical Sample Testing by CCHFV-RT-LAMP

**Figure 3:**
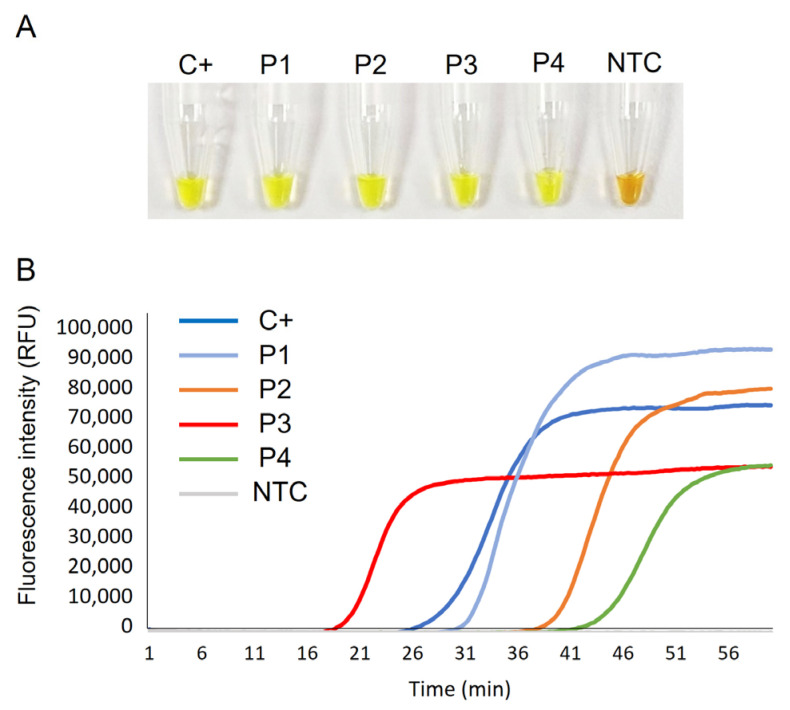
Clinical sample testing by CCHFV-RT-LAMP. (**A**) Colorimetric RT-LAMP results by SYBR green end-point addition. (**B**) Real-time RT-LAMP results showing EvaGreen 20× fluorescence signal over time. C+, RNA isolate from an infected patient used as positive control (P5); P1–P4, RNA isolates from patients with CCHF; NTC, non-template control. RFU, relative fluorescence units.

Figure 3 shows the detection of CCHFV RNA in clinical samples by colorimetric (Figure 3A) and real-time CCHFV-RT-LAMP (Figure 3B). A positive result was obtained in all samples tested; negative control (NTC) did not amplify. The RNA isolate used as positive control (P5; C+) was amplified with identical Tp value as in the set-up test (24 min) and with the same relative fluorescence units (RFU) values (approx. 75,000 RFU) in fluorescence intensity measurements. Real-time RT-LAMP detected CCHFV RNA in patients’ samples with the following Tp and RFU values, respectively: P1 (29 min; 90,000 RFU); P2 (38 min; 80,000 RFU); P3 (18 min; 50,000 RFU); P4 (41 min; 50,000 FRU).

**Figure 4:**
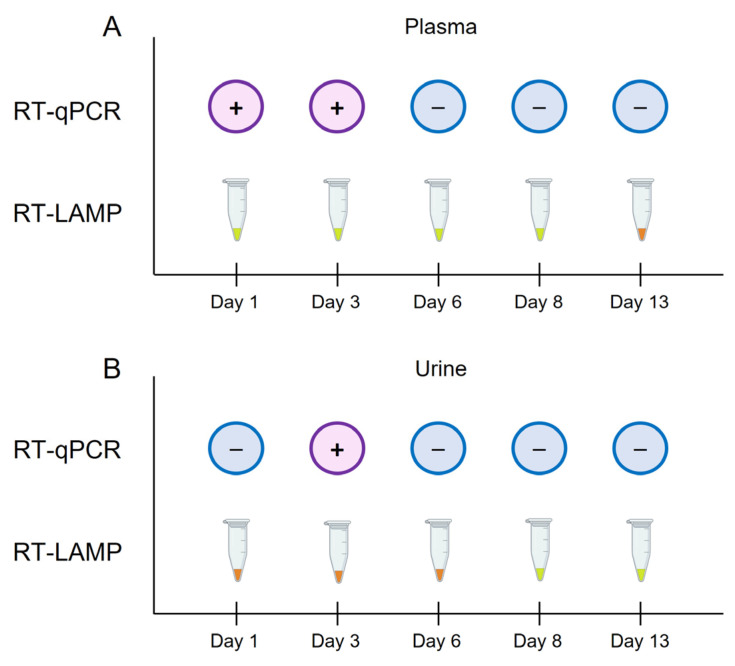
Schematic representation of the comparison of the results obtained by RT-qPCR and colorimetric RT-LAMP in plasma and urine samples from patient P4 over days 1, 3, 6, 8 and 13, during the patient’s admission at hospital. (**A**) Results obtained by RT-qPCR vs. RT-LAMP in plasma samples. (**B**) Results obtained by RT-qPCR vs. RT-LAMP in urine samples. For RT-qPCR, the + symbol in a purple circle indicates a positive result; the − symbol in a blue circle indicates a negative result. For colorimetric RT-LAMP, the results were visually detected by colour change: green/positive; orange/negative.

In addition, plasma and urine samples from patient P4 were analysed by both RT-qPCR and colorimetric RT-LAMP over several days (days 1, 3, 6, 8 and 13) during the patient’s admission at Hospital Universitario de Salamanca (HUS), Salamanca, Spain **(**Figure 4). In plasma samples, RT-qPCR positive results were obtained at days 1 and 3, whereas colorimetric RT-LAMP was able to detect CCHFV RNA at days 1, 3, 6 and 8 (Figure 4A). In urine samples, only an RT-qPCR result was obtained at day 3, whereas colorimetric RT-LAMP detected CCHFV RNA at days 8 and 13 of convalescence (Figure 4B).

### 2.4. DNA Sequencing and Phylogenetic Analysis

**Figure 5:**
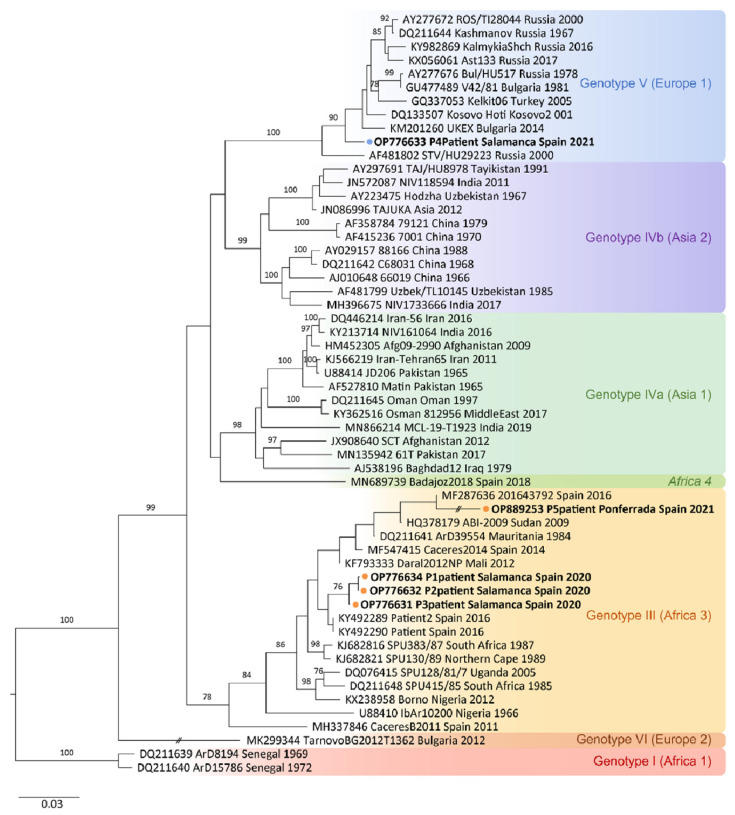
Phylogenetic tree showing the positions of the 51 S-segment sequences used for the CCHFV-RT-LAMP design and the newly identified CCHFV RNA sequences from patients’ samples included in this study. Phylogenetic tree constructed using the neighbour-joining method based on partial (200 nt) sequences of the virus small segment. Numbers in branches indicate bootstrap values for the groups; values < 75 are not shown. An interrupted branch (//) indicates its length has been reduced to half. Dots (orange for P1, P2, P3 and P5; blue for P4) and bold letters indicate patients analysed in this study and named by GenBank accession number, locality sampling site, geographic origin, and sampling year; other sequences are indicated by GenBank accession number, strain, geographic origin, and sampling year. Genotypes are indicated by roman numerals and clade nomenclature indicated in brackets, using nomenclature published by Carrol et al. [9] and Chamberlain et al. [55]: I, West Africa (Africa 1); III, South and West Africa (Africa 3); IV, Middle East/Asia, divided in two groups corresponding to groups Asia1 y Asia 2; V, Europe/Turkey (Europe 1); VI, Greece (Europe 2). Italics indicate new lineage, Africa 4 described by Negredo et al. [19]. Scale bar indicates nucleotide substitution per site.

The CCHFV S-segment sequences obtained from patients P1 (219 bp), P2 (220 bp) and P3 (202 bp) showed 98%, 99% and 99% identity, respectively, with sequence HQ378179 (Sudan AB1-2009 strain) from Sudan. The three sequences fall in reported African III genotype. The CCHFV S-segment obtained from patient P4 (204 bp) showed 98% identity with sequence DK133507 (Kosovo Hoti strain) from Kosovo. The phylogenetic analysis grouped this sequence in the European V genotype. As mentioned above, S-segment sequence of 212 bp from patient P5 showed partial 98.08% identity with Daral 2012 strain (GenBank KF793333) of African origin (Mali) belonging to African genotype III. All the sequences obtained in this study were deposited in GenBank under the following accession numbers: P1, OP776634; P2, OP776632; P3, OP776631; P4, OP776633; P5, OP889253. The phylogenetic analysis of CCHFV S-segment sequences is shown in Figure 5.

## 3. Discussion

At present, CCHFV has been detected circulating in tick species and animals in western, southern, and central areas of Spain, showing an increasing spread of the virus to south-western Europe and, consequently, an increase in the risk of cases of CCHF. In recent years, 12 autochthonous cases have been reported in Spain, including four fatal cases. The absence of effective treatments and vaccines against CCHF, together with the wide spread of the virus, the mode of transmission and the severity of the disease, make CCHF a major threat to global health [56]. Because of this, a rapid and sensitive diagnosis of the disease is essential. Nucleic acid amplification tests (mainly RT-qPCR) combined with serological methods are the most applied for diagnosis of CCHF in reference laboratories [57,58]. However, due to the high genetic diversity of CCHFV, more studies are needed to evaluate the sensitivity of serological methods and the capability of RT-qPCR protocols to detect all known virus lineages [32]. Despite there being several commercial tests for PCR and serology available, most international labs use in-house assays, probably due to an investment in tests developed from regional CCHV strains and because commercial tests are expensive or not available to international customers [58]. Moreover, RT-qPCR methods have the disadvantages of requiring very expensive reagents and equipment and technically qualified personnel to operate them. Therefore, the use of a rapid, sensitive and easy-to-perform isothermal molecular test such as RT-LAMP would be very useful in low-resource settings or field diagnostics in rural and remote regions affected by CCHFV.

Considering the high genetic variability and reassortment due to the trisegmented genome (S, M, L) of CCHFV, primer design for molecular detection of CCHFV becomes a challenge. RT-PCR assays tend to be lineage-specific for regional circulating strains, so there is an urgent need for pan-lineage sensitive diagnostics [56]. Hence, in our study, with the aim of improving molecular diagnostics of CCHF, a novel RT-LAMP assay was developed to detect RNA from all potential lineages of CCHFV. Furthermore, its performance was evaluated in clinical samples from patients with CCHF and compared with that of RT-qPCR in plasma and urine samples.

To design the set of primers for our RT-LAMP, a total of 51 available sequences of the S segment of the CCHFV genome from diverse virus strains or isolates with different geographical origin were retrieved from databases. We used this target because it is the most conserved region of the CCHFV genome across geographical isolates [58]. A consecutive multiple ‘intra and inter-geographical’ alignment of the selected sequences generated a 1672 bp global consensus sequence that showed 92.8% identity with the sequence of the S segment of CCHFV strain C-68031 (GenBank DQ211642), with origin in China [59]. This global consensus sequence was selected in silico to design a set of primers, and a refinement strategy with ‘wobble’ bases was applied to compensate for target global consensus sequence variability in order to detect the highest number of CCHFV variants. For previous RT-LAMP primer designs to target the S segment, other authors used only S-segment sequences specific for regional circulating strains from Sudan [52] or Russia [53], thus theoretically reducing the possibility of detecting other likely circulating genotypes.

Initially, we tried to establish the proper operation, sensitivity, and specificity of both RT-PCR (using the outer primers F3-B3) and colorimetric RT-LAMP assay (using the set of four primers) in targeting CCHFV RNA of a patient isolate used as amplification control (P5; C+). RT-PCR F3-B3 yielded the expected 212 bp in length fragment, thus confirming the correct target, and both molecular assays were specific for CCHFV because no cross-reactivity was observed when RNA from other viruses, including haemorrhagic viruses such as Zaire Ebola virus, Sudan Ebola virus and Lassa virus were used as template.

Regarding sensitivity, the limit of detection using colorimetric RT-LAMP (50 fg/µL) resulted up to 10^3^ times lower than that obtained by RT-PCR F3-B3 (50 pg/µL). The higher sensitivity usually obtained by LAMP assays compared to PCR reactions is well known [60]. Furthermore, these results are in line with those of Osman et al. [52] in the development of RT-LAMP for the detection of CCHFV, in which the sensitivity obtained by RT-PCR using the outer primers was 100 times lower than that obtained by RT-LAMP using the set of four primers. It should be noted that the sensitivity of our RT-LAMP was lower than that obtained by Osman et al. [52] for Sudanese strains of CCHFV. This may be due to the fact that our RT-LAMP design was based on degenerate primers, which could potentially decrease the sensitivity of the assay by forming mismatches with templates. The major challenge for virus detection by LAMP is the high genetic diversity of some viral genomes, which exist in the forms of genotypes, subtypes, strains, and/or quasi-species [61,62,63]. Although a strategy of combining multiple degenerate primers together has been developed for broad-spectrum detection of various genotypes of genetically diverse viruses (e.g., DENV, HIV-1, influenza A enteroviruses), the low detection efficiency of LAMP for several viral variants remains unresolved [64]. As it is virtually impossible to detect all variants in one LAMP assay, underestimation of viral load may occur using the RT-LAMP method, especially for highly variable RNA viruses [64,65]. Increasing degeneracy raises the possibility of decreasing efficiency, but increases the likelihood of finding unknown divergent variants of a sequence family. This dual behaviour must be taken into account, and the design of degenerate primers must be a compromise between specificity and coverage (sensitivity). With this in mind, our RT-LAMP to detect CCHFV achieved very satisfactory sensitivity (50 fg/µL) while potentially allowing the detection of a larger number of viral variants. It is important to note here that the sensitivity of the assay could possibly have been increased if a temperature gradient had been tested in our RT-LAMP. Future trials will be directed towards such tests.

The sensitivity and specificity of colorimetric RT-LAMP were corroborated in real-time testing by performing the assay on a portable device with fluorescence readout. Subsequently, the efficacy of both assays was evaluated using RNA isolates from CCHF patients confirmed by RT-qPCR. All samples were successfully amplified showing different time to positivity (Tp) and relative fluorescence unit (RFU) values. Unfortunately, as we did not know the cycle threshold (Ct) values of RT-qPCR and did not have a standard RNA quantification to use in the RT-LAMP assay, it was not possible to perform a comparison of speed and sensitivity between RT-qPCR and RT-LAMP in the analysis of patient samples. Most authors use as threshold in LAMP assays the Tp value to indicate the occurrence of a positive or negative result by similarity, although not exactly, to the Ct of a qPCR. It has been reported that RT-qPCR amplification Ct values and RT-LAMP amplification Tp values are equivalent for a given amplified viral RNA concentration [66]. In RT-qPCR studies, a correlation between CCHFV RNA load and disease progression has been reported, with a worse clinical outcome in patients with a high viral load and viral clearance being considered the most important indicator of survival in cases of CCHF [57,67,68]. Interestingly, in our study, the only patient with a fatal outcome (patient P3) showed the shortest time to positivity (Tp = 18 min) in real-time RT-LAMP, so it was probably the sample with the highest viral load. In our previous experience working on RT-LAMP amplification of RNA viruses, such as SARS-CoV-2, the Tp values in positive samples were even lower than the Ct values obtained in RT-qPCR, clearly indicating the high efficiency of the RT-LAMP assay in viral RNA amplification [69]. Notwithstanding the above, we are aware of the limitation in this study of not having real-time PCR Ct values available, so it was not possible to calculate the corresponding standard lines for the calculation of RNA in copies per reaction.

Regarding genetic variability, sequencing of amplicons obtained by RT-qPCR from patients P1, P2, P3, and P4 enabled us to identify different genotypes on the basis of their S-segment sequences. On one hand, S-segment sequences from patients P1, P2 and P3 were found to belong to the African III genotype. In Spain, it is supposed that the African III genotype was introduced by migratory birds from West Africa [70] and it has been demonstrated as the most common genotype circulating in ticks [11,12,13,14]. Of interest, genotype III was also demonstrated in the first autochthonous CCHF case reported in Spain [10]. On the other hand, the phylogenetic analysis grouped the CCHFV S-segment sequence obtained from patient P4 with viruses of the European V genotype, a recently described genotype in ticks in Spain [14] and more recently in a retrospective case of a Spanish patient with CCHF, suggesting that CCHFV is an identifiable cause of febrile illness of unknown origin and a possible establishment of a transmission cycle of CCHFV genotype V in this country [20]. The S-segment sequence of 212 bp obtained from patient P5 (C+) by conventional RT-PCR using outer primers F3-B3 was also found to belong to African genotype III. Therefore, according to the sequencing results, our RT-LAMP allowed for the amplification of two different CCHFV genotypes in patients’ samples: the African III genotype and the European V genotype. This is evidence that RT-LAMP based on degenerated primers is well designed to detect different CCHFV genotypes circulating in Spain.

With regard to specimens for molecular detection of CCHFV, the available data are limited to a small number of patients and detailed comparisons of sensitivity between molecular methods for diagnosis in different sample types are not possible, in particular of urine versus blood samples (serum, plasma or whole blood). In our study, we had the opportunity to compare RT-qPCR and colorimetric RT-LAMP analysis of plasma and urine samples from patient P4 collected at days 1 to 13 during admission at the University Hospital of Salamanca. In plasma samples, RT-qPCR was positive on the first and third days, whereas RT-LAMP remained positive up to day 8. It has been reported that CCHFV RNA can be detected by RT-qPCR up to the 18th day of illness in serum samples, with most successful results during the first 5 days after onset of symptoms [32]. In some cases, it has even been detected up to 36 days of infection [71]. However, to date, no results have been reported for the molecular detection of CCHFV RNA in plasma samples during the course of infection. According to our results, RT-LAMP should be more sensitive than RT-qPCR in detecting viral RNA in plasma samples from CCHFV patients. In urine samples, only an RT-qPCR result was obtained at day after patient admission; however, our RT-LAMP detected CCHFV RNA at day 8 and day 13 of convalescence. Previous studies have also reported the persistence of viral RNA in urine samples despite serum clearance [71,72] and that viral loads in urine samples are similar to those encountered in blood [73]. To our knowledge, our work is the first report of detection of CCHFV RNA by RT-LAMP in urine of a patient. As highlighted in other studies, the presence of CCHFV RNA in urine is an important observation for viral pathogenesis and transmission and may have implications for public health. In addition to standard precautions, additional contact precautions for CCHF inpatients may be considered, even when haemorrhage is not present [71,72]. Nonetheless, further studies are needed to determine the diagnostic applicability of urine, the frequency of urinary excretion and its duration during convalescence. The limited number of cases of CCHFV in Spain and the difficulty in obtaining more clinical samples for the study should be highlighted. In addition, it should be noted that better access to clinical samples of CCHFV patients for diagnostic validation would help to accelerate the development of new tests.

## 4. Materials and Methods

### 4.1. Ethics Statement

The study protocol was approved by the Clinical Research Ethics Committee of Investigation with Drugs of Hospital Universitario de Salamanca (HUS), Salamanca, Spain (CEIMC PI 9109/2017). All procedures described were carried out in accordance with the ethical standards described in the Revised Declaration of Helsinki of 2013. All data from patients were anonymised.

### 4.2. CCHFV RNA-Positive Control and Patients’ RNA Samples

In this study, five RNA samples obtained from five patients diagnosed with CCHF in Spain were used. Four cases (patients P1, P2, P3, and P4) were diagnosed at Hospital Universitario de Salamanca (HUS), Salamanca, Spain in 2020 to 2021. Another case (P5) was diagnosed at Hospital del Bierzo (Ponferrada, León, Spain) in June 2021. At the time, the diagnosis of the five CCHF cases was confirmed by PCR methods designed to amplify two different targets of the CCHF viral genome at the Arbovirus Laboratory, National Microbiology Center, Institute of Health Carlos III, Madrid, Spain, as described by Negredo et al. [10]. The main data of patients included in the study are shown in Table 1.

The availability of clinical samples of patients diagnosed of CCHF is very limited. Thus, the first RNA isolate we had access to was from P5, so it was this sample that we used to assess the operation of the RT-LAMP method for amplification of CCHFV RNA (hereafter, positive control; C+). RNA was measured by using a NanoDrop ND-1000 spectrophotometer (NanoDrop Technologies, Wilmington, NC, USA) and then diluted with RNase-free water to a final concentration of 5 ng/µL. Serial 10-fold dilutions of the C+ in RNase-free water (ranging from 1× to 10^−6^) were prepared and stored at −80 °C until use. The RNA sample thus prepared was used as C+ in all amplification reactions and for assessing sensitivity of molecular assays.

Later, we were also provided with RNA samples from patients P1–P4 and they were then included in the study. Additionally, from P4 we were provided with RNA from both plasma and urine samples collected on days 1, 3, 6, 8 and 13 during their admission to the HUS. In this case, plasma and urine samples were handled and inactivated in biosafety level 3 (BSL-3) laboratory (I + D + I Building Facilities, University of Salamanca) so that they could then be handled at lab under BSL-1 conditions. Subsequent RNA extraction was carried out using the NZY Viral RNA Isolation Kit (NZYTECH, Lisbon, Portugal) following manufacturer instructions. All RNA samples were kept at −80 °C until further analysis.

### 4.3. Design of Reverse-Transcription Loop-Mediated Isothermal Amplification Assay (RT-LAMP) for CCHFV

#### Sequence Selection and Primer Design for CCHFV-RT-LAMP

**Figure 6:**
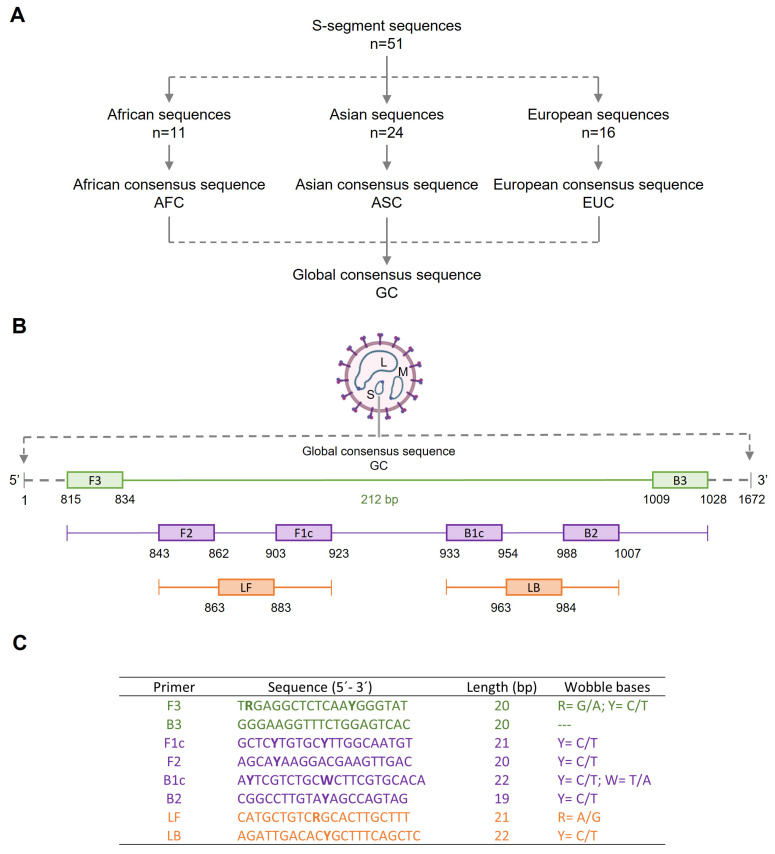
Schematic representation of the process for the design of reverse-transcription loop-mediated isothermal amplification assay (RT-LAMP) for CCHFV. (**A**) Outline for global consensus (GC) sequence selection based on several linear single-strand RNA S-segments from CCHFV. (**B**) Outline of location of set of primers in the partial GC sequence used as target. (**C**). Sequences of the RT-LAMP primers finally selected. F3, forward primer; B3, backward primer: F1c + F2 sequences: FIP, forward inner primer; B1c + B2 sequences: BIP, backward inner primer; LF, loop forward primer; LB, loop backward primer. Bold letter codes (R, Y or W) are used to represent the combination of two different nucleotide phosphoramidites blended at equimolar ratios prior to coupling at that position in the sequence.

Sequence selection. Segment S of CCHFV was preferred as the amplification target for the primer design because it is the one on which most phylogenetic analysis studies have focused. A total of 51 sequences corresponding to a linear single-strand RNA complete or partial sequence in the CCHFV segment S were selected and retrieved from GenBank to be used for the design of primers. The 51 sequences were divided into three groups according to the geographic origin of the isolates, as follows: Africa (*n* = 11), Asia (*n* = 24) and Europe (*n* = 16) (see Table 2). Subsequently, a multiple alignment using ClustalW [74] into each group resulted in a consensus sequence, thus obtaining three different ‘geographical’ consensus sequences referred to as ‘African consensus’ (AFC), ‘Asian consensus’ (ASC) and ‘European consensus’ (EUC). Next, the AFC, ASC and EUC sequences were aligned with each other using ClustalW and a 1672 base pair (bp) global consensus (GC) sequence was generated. A scheme of the process of obtaining the GC sequence is shown in Figure 6A. Finally, a BLASTN search and alignment analysis [75] on the NCBI database resulted in a final GC sequence with 92.8% similarity to the sequence reported for the S segment for the CCHFV strain C-68031 (GenBank DQ211642). In addition, no regions of similarity between this GC sequence and other sequences reported for humans or possible human pathogens were detected.

Primer design. RT-LAMP primers to amplify RNA of CCHFV were designed based on the 1672 bp GC sequence using the PrimerExplorer V5 software program (https://primerexplorer.jp/e/, accessed on 20 January 2022). A number of potential LAMP primer sets were generated and further refinement in design was manually developed following the instructions described in “A guide to LAMP primer designing” [76]. The locations and target sequence are shown in Figure 6B. When comparing multiple sequences in generating the GC sequence, we found that the alignment sometimes revealed insufficient consensus to accommodate a single oligonucleotide for use as a primer. Occasionally, only one or two nucleotides did not match. To design primers for these regions, we opted to introduce a degenerate site, or ‘wobble’, to compensate for target sequence variability. The set of primers finally selected with their corresponding wobble bases is indicated in Figure 6C.

**Table 2:** Crimean–Congo haemorrhagic fever virus S-segment sequences retrieved from GenBank used in this study grouped by geographical origin of the strains/isolates to obtain a global consensus sequence for designing a set of primers for reverse-transcription loop-mediated isothermal assay for the detection of Crimean–Congo haemorrhagic virus RNA. NA, not available. “*” means: Not identified.

Origin (*n*)	Genbank Accession	Strain/Isolate	Location, Isolation Year	Source of Isolate	Reference
African sequences (*n* = 11)	DQ076415	SPU128/81/7	Uganda (Semunya)	Tick *	[55]
	DQ211639	ArD8194	Senegal, 1969	*H. truncatum*	[59]
	DQ211640	ArD15786	Senegal, 1972	Goat	[59]
	DQ211641	ArD39554	Mauritania, 1984	*H. marginatum*	[59]
	DQ211648	SPU415/85	South Africa, 1985	Human	[59]
	HQ378179	AB1-2009	Sudan (Abyei), 2009	Human	[23]
	KF793333	Daral 2012 NP	Mali (Daral), 2012	*Hyalomma*	[77]
	KJ682816	SPU383/87	South Africa, 1987	Human	[78]
	KJ682821	SPU130/89	Northern Cape, 1989	Human	[78]
	KX238958	NA	Nigeria (Borno), 2012	Human	NA
	U88410	IbAr10200	Nigeria, 1966	NA	NA
Asian Sequences (*n* = 24)	AF358784	79121	China, 1979	NA	NA
	AF415236	7001	China, 1970	NA	NA
	AF481799	Uzbek/TI10145	Uzbekistan, 1985	Human	[79]
	AF527810	Matin	Pakistan, 1965	NA	NA
	AJ010648	66019	China, 1966	NA	[80]
	AJ538196	Baghdad-12	Iraq, 1979	Human	[80]
	AY029157	88166	China, 1988	NA	NA
	AY223475	Hodzha	Uzbekistan, 1967	Human	[80]
	AY297691	TAJ/HU8978	Tajikistan, 1991	Human	[81]
	DQ211642	C-68031	China, 1968	Sheep	[59]
	DQ211645	Oman	Oman, 1997	Human	[59]
	DQ446214	Iran-56	Iran, 2017	Human	NA
	GQ337053	Turkey-Kelkit06	Turkey, 2005	Human	[82]
	HM452305	Afg09-2990	Afghanistan, 2009	Human	[83]
	JN086996	TAJUK	Asia, 2012	NA	[28]
	JN572087	NIV118594	India, 2011	*H. antolicum*	[84]
	JX908640	SCT	Afghanistan, 2012	Human	[85]
	KJ566219	Iran-Tehran65	Iran, 2011	Human	[86]
	KY213714	NIV161064	India, 2016	Human	[87]
	KY362516	Oman 812956	Middle Eastern, 2017	Human	NA
	MH396675	NIV1733666	India	Human	NA
	MN135942	61T/Pakistan	Pakistan, 2017	Tick	[88]
	U88414	JD 206	Pakistan, 1965	NA	NA
	MN866214	MCL-19-T-1923	India, 2019	Human	[89]
European sequences (*n* = 16)	MH337846	Cáceres/B 2011	Spain, 2011	*H. lusitanicum*	[13]
	KX056061	Ast133	Russia, 2017	Human	NA
	KY982869	Kalmykia_Shch_2_2016	Russia, 2016	Human	[90]
	MK299344	Malko Tarnovo-BG2012-T1362	Bulgaria, 2012	*R. bursa*	[91]
	MN689739	Badajoz 2018	Spain, 2018	Human	NA
	MF547415	Cáceres 2014	Spain, 2014	*H. lusitanicum*	[12]
	DQ133507	Kosovo Hoti	Kosovo, 2001	Human	[26]
	KM201260	UK ex	Bulgaria, 2014	Human	[92]
	GU477489	V42/81	Bulgaria, 1981	Human	[93]
	AY277676	NA	Russia	Human	NA
	AY277672	ROS/TI28044	Russia, 2000	*H. marginatum*	NA
	DQ211644	Kashmanov	Russia, 1967	Human	[59]
	AF481802	STV/HU29223	Russia, 2000	Human	[79]
	KY492290	patient1	Spain, 2016	Human	[10]
	KY492289	patient2	Spain, 2016	Human	[10]
	MF287636	201643792	Spain, 2016	Human	[8]

### 4.4. RT-PCR Using Outer Primers F3 and B3

The outer RT-LAMP primers F3 and B3 were firstly tested for the detection of CCHFV RNA by RT-qPCR to verify whether the expected in silico 212 bp GC sequence was amplified. For RT-qPCR, the one-step NZY RT-qPCR green kit ROX (Nzytech, Lda., Lisbon, Portugal) was used following manufacturer instructions. The reactions were carried out in 20 µL reaction mixture containing 10 µL of ROX master mix (2x), 0.8 µL 10 µM forward primer (F3) and 10 µM reverse primer (B3), 0.8 µL NZYRT mix, 2.6 µL nuclease-free water and 5 µL purified RNA template (P5; C+). The cycling parameters were as follows: reverse transcription for 20 min at 50 °C, for 15 min at 95 °C as polymerase activation, 40 cycles of 15 s of denaturation at 95 °C, for 1 min at 60 °C as annealing/extension. The reactions were performed in a PCRmax Eco48 real-time PCR system (PCRmax, Beacon Road, Stone, Staffordshire, UK). Subsequently, 3–5 µL of RT-qPCR amplicons was subjected to 1.5% agarose gel electrophoresis using gel loading buffer with GreenSafe Premium DNA stain (NZYtech, Lda., Lisbon, Portugal; MB13201) and visualised under UV light. Negative controls (RNase-free water instead RNA template) were included in all trials.

The sensitivity of the RT-qPCR using the outer primers F3 and B3 was also assayed to establish the detection limit of CCHFV RNA using those 10-fold serial dilutions of C+ prepared mentioned above.

### 4.5. Establishing the RT-LAMP Assay for CCHFV Detection

#### 4.5.1. Conventional Colorimetric CCHFV-RT-LAMP

The set of primers designed was first evaluated for colorimetric visualisation in RT-LAMP reactions mixtures (25 µL) containing: 1.6 µM FIP/BIP primers, 0.2 µM F3/B3 primers, 0.4 µM LF/LB primers, 0.4 µM of dNTPs, 6 mM MgSO_4_, 1X Isothermal Amplification Buffer, 1 µL *Bst* polymerase 2.0 WarmStart (New England Biolabs Ltd., Ipswich, MA, USA) and 0.5 µL *RTx* Reverse Transcriptase (New England Biolabs Ltd., Ipswich, MA, USA), with 3 µL of template RNA (C+, for positive control; RNase-free water instead RNA for negative control). The RT-LAMP reactions were performed in 0.5-mL micro-centrifuge tubes by incubation in a heating block at 63 °C for 60 min followed at 80 °C for 10 min to stop the reaction. The RT-LAMP amplification results were visually inspected by adding 2 µL of 1:10 diluted 10,000× *g* concentration fluorescent dye SYBR Green I (Invitrogen, Waltham, MA, USA), in each reaction tube. Green fluorescence was observed in the successful RT-LAMP reaction and original orange in the negative reaction. The tubes were briefly centrifuged and carefully opened before adding the dye to avoid possible cross-contamination with amplified products.

#### 4.5.2. Real-Time CCHFV-RT-LAMP

Real-time CCHFV-RT-LAMP was also attempted in a one-step RT-LAMP reaction using the same set of primers and mixtures as the conventional colorimetric assay, but in this case, with the addition of 0.40 µL/tube of EvaGreen 20× in water (Biotium, San Francisco, CA, USA) to the reaction mix before the reaction started to monitor the fluorescence over time. The RT-LAMP was carried out in 8-tube Genie Strips on a portable Genie III instrument (OPTIGENE Ltd., Horsham, UK) at 63 °C for 70–80 min followed by 10 min at 80 °C to stop the reaction. RT-LAMP assays were also performed with an initial step at 50 °C for 15 min to facilitate the reverse transcription followed by 50 min at 63 °C, and then heated at 80 °C for 10 min to stop the reaction.

#### 4.5.3. Sensitivity and Specificity of CCHFV-RT-LAMP

Sensitivity and specificity were assessed in both colorimetric and real-time CCHFV-RT-LAMP. The analytical sensitivity of the set of primers in the detection of CCHFV was evaluated using the 10-fold serial dilutions of the C+ mentioned above. To determine the specificity, a BLAST local search and alignment analysis was carried out firstly in silico in GenBank online database (https://blast.ncbi.nlm.nih.gov, accessed on 1 March 2022) against nucleotide sequences for other human-infecting viruses, such as Zaire Ebola virus (taxid 186538), Sudan Ebola virus (taxid 186540), Lassa mammarenavirus (taxid 11620), Marburg Marburgvirus (taxid 11269), yellow fever virus (taxid 11089), Rift Valley fever virus (taxid 11588), West Nile virus (taxid 11082), dengue virus (taxid 12637) and chikungunya virus (taxid 37124). In addition, the set of primers designed were cross-tested for specificity in both colorimetric and real time CCHFV RT-LAMP assays against a panel of 8 RNA isolates of several other viruses, including haemorrhagic viruses: Zaire Ebola virus (ZEBOV), Sudan Ebola virus (SEBOV) and Lassa virus (LSSV) (kindly provided by the National Center of Microbiology, Institute of Health Carlos III, Majadahonda, Madrid, Spain), and also respiratory RNA viruses: respiratory syncytial virus A (RSVA), respiratory syncytial virus B (RSVB), coronavirus NL63 (NL63), coronavirus OC43 (OC43) and influenza A H1 (AH1) (kindly provided by the Laboratory of the National Influenza Centre of Valladolid, University Clinical Hospital of Valladolid, Valladolid, Castilla y León, Spain).

#### 4.5.4. Clinical Samples Testing by CCHFV-RT-LAMP

Based on the analysis with RT-qPCR, the RNA isolates from CCHF patients P1, P2, P3 and P4, were analysed by colorimetric and real-time CCHFV-RT-LAMP assays. Additionally, RNA isolates from plasma and urine samples from patient P4 were analysed by colorimetric CCHFV-RT-LAMP.

### 4.6. DNA Sequencing

An aliquot of each RNA isolates stored at −80 °C obtained from plasma samples from patients P1–P4 were sent to DNA Sequencing Service (NUCLEUS), University of Salamanca, Salamanca, Spain, for sequencing. A nested reverse-transcription PCR (nested RT-PCR) was performed as described elsewhere to amplify the 123–764 region in the first amplification and the 450–674 region in the second amplification in the S segment of CCHFV [10]. Subsequently, DNA amplified was purified and the double-stranded DNA was directly sequenced with the same second primers set used in nested RT-PCR by the Sanger chain-termination method and the 3500 Series Genetic Analyzer (Applied Biosystems; ThermoFisher Scientific, Waltham, MA, USA). Consensus sequences of each segment were assembled and analysed using BioEdit 7.2 Sequence Alignment Editor software [94]. The sequences obtained in this study were deposited to GenBank and an accession number was provided for all submitted sequences.

The 212 bp amplicon obtained from RNA-positive control (P5; C+) in RT-PCR using the outer primers F3-B3 designed in this study was purified using NZY Gelpure (Nzytech, Lisbon, Portugal) according to the manufacturer protocol and sent refrigerated for bidirectional Sanger sequencing to DNA Sequencing Service (NUCLEUS), University of Salamanca, Salamanca, Spain, to verify target amplification. For sequencing, the same outer F3-B3 primers were used. The sequence obtained was subsequently examined using BioEdit Sequence Alignment Editor software [94] and a BLASTN was performed to compare the sequence with those available in GenBank nucleotide database. The sequence obtained was also deposited to GenBank.

### 4.7. Phylogenetic Analysis

The phylogenetic analysis was carried out using ClustalW (https://www.genome.jp/tools-bin/clustalw, accessed on 21 October 2022) to align the sequences of the CCHFV S segment obtained from patients’ samples in this study and those sequences available in GenBank for comparison. A Tamura–Nei parameter model was selected to construct the phylogenetic tree based on the 51 S-fragment sequences of CCHFV selected from the database. A phylogenetic tree was generated by the neighbour-joining method using Geneious Tree Builder software 2022 [95] with 1000 replicates bootstrap testing and was edited using Adobe^®^ Illustrator CS4.

## 5. Conclusions

In summary, we have developed a novel, rapid, specific and sensitive RT-LAMP test that allows the detection of different CCHFV genotypes in clinical samples. This pan-CCHFV RT-LAMP has proven to be effective in detecting viral RNA in plasma and urine samples. Moreover, it can be simply performed as a single-tube isothermal colorimetric method without any expensive equipment requirement and in a real-time portable platform, thus bringing molecular diagnostics closer to rural or resource-poor areas. As more and more samples are obtained from ticks, animals and humans in endemic regions and the virus continues to evolve over time, our new pan-CCHFV RT-LAMP could be a promising molecular tool to detect as many CCHFV variants as possible. However, more research is needed for validation of the pan-CCHFV RT-LAMP assay using a larger number of samples and evaluation of more genotypes. The use of environmental samples, such as the detection of CCHFV in ticks, would be useful to fully validate the RT-LAMP assay. Further work is needed to develop new, efficient and easily applicable molecular methods to diagnose CCHF.

## Figures and Tables

**Table 1:** Main data of Crimean–Congo haemorrhagic fever patients included in this study. P1–P5, patient samples; C+, positive control.

	P1	P2	P3	P4	P5 (C+)
Sex	Male	Male	Male	Male	Female
Age (years)	70	54	69	59	30
Date	June 2020	July 2020	August 2020	April 2021	June 2021
Tick bite	Yes	Yes	Yes	Yes	Yes
Habitat	Rural	Rural	Rural	Rural	Rural
Fever	Yes	Yes	Yes	Yes	Yes
Any bleeding symptomatology	Yes	No	Yes	No	Yes
*Exitus letalis*	No	No	Yes	No	No

## Data Availability

Not applicable.
